# Watercore Pear Fruit Respiration Changed and Accumulated γ-Aminobutyric Acid (GABA) in Response to Inner Hypoxia Stress

**DOI:** 10.3390/genes13060977

**Published:** 2022-05-30

**Authors:** Xiao Liu, Dong-He Liu, Tao Chen, Jing Zhang, Chun-Lei Wang

**Affiliations:** School of Horticulture and Plant Protection, Yangzhou University, Yangzhou 225009, China; liuxiao@yzu.edu.cn (X.L.); ldh111402@163.com (D.-H.L.); ct18356125761@163.com (T.C.); zhangj45@yzu.edu.cn (J.Z.)

**Keywords:** watercore, pear, TCA cycle, γ-aminobutyric acid

## Abstract

Watercore is a physiological disorder which often occurs on the pear fruit and the excessive accumulation of sorbitol in fruit intercellular space is considered to be an important cause of watercore. Our previous studies found that the metabolic disorder of sugars may lead to hypoxia stress and disturb respiration, resulting in aggravated fruit rot and the formation of bitter substances. However, the further changes of respiration and the fruit response mechanism are not well understood. A comprehensive transcriptome analysis of ‘Akibae’ pear watercore fruit was performed in this study. The transcriptome results revealed the hypoxia stress significantly induced the expression of several key enzymes in the TCA cycle and may lead to the accumulation of succinic acid in watercore fruit. The glycolytic pathway was also significantly enhanced in watercore fruit. Moreover, the γ-aminobutyric acid (GABA) synthesis related genes, glutamate decarboxylase (GAD) genes and polyamine oxidase (PAO) genes, which associated with the GABA shunt and the polyamine degradation pathway were significantly upregulated. In addition, the *PpGAD1* transcript level increased significantly along with the increase of GAD activity and GABA content in the watercore fruit. Above all, these findings suggested that the hypoxic response was marked by a significant increase of the hypoxia-inducible metabolites succinic acid and GABA and that *PpGAD1* may play a key role in response to watercore by controlling the GABA synthesis.

## 1. Introduction

Watercore is a physiological disorder in pear and apple fruit which can lead to economic losses. Symptoms of the watercore fruit flesh include translucent, watery tissue, often accompanied by browning [1]. Watercore fruits are common in well-ripened fruit and the strong interactions between these characteristics make it difficult to clarify the mechanisms involved in the disorder. The disorder not only appears when the fruit is attached to the tree, but also progress after harvest, distinguishing it from the problem of internal breakdown or browning in pear that occurs during storage [2]. 

The cause of higher sorbitol accumulation is attributed to lower sorbitol transporter expression in watercore affected tissue [3]. Instead of transporting sorbitol into fruit storage parenchyma, it accumulates in intercellular spaces, and thus decreases intercellular space volume, which we speculate it can lead to hypoxia stress and ultimately, changing the internal flavor [4,5]. Plant adaptation to hypoxia is quiescence strategy. In general, quiescent plants down-regulate the oxygen-consuming and related metabolic pathways, and maintain a minimum mitochondrial respiration to keep the plant alive [6,7]. Typically, the trichloroacetic acid cycle (TCA) are inhibited by hypoxia, resulting in a sharp reduction of respiratory rate [8]. Plant cells depend on the energy produced by glycolytic pathway via substrate-level phosphorylation under hypoxia. The glycolytic pathway produces two mole ATP and two mole pyruvate per mole hexose while reducing NAD^+^ to NADH. The NAD^+^ is regenerated when pyruvate is reduced into lactate by lactate dehydrogenase (LDH), or into ethanol by pyruvate decarboxylase (PDC) and alcohol dehydrogenase (ADH) [6,9].

The accumulation of γ-aminobutyric acid (GABA) is a metabolic response of plant systems to stress such as salinity, hypoxia, drought, heat, and chilling, is has a positive correlation between GABA content and the activities of antioxidant enzyme, which plays an important role in plant recovery [10,11,12,13,14]. There are two main anabolic pathways of GABA in plants, namely the GABA shunt with glutamate as the precursor and the polyamine degradation pathway with polyamine as substrate, among which GABA shunt plays more important roles. In the GABA shunt, glutamate is irreversibly catalyzed to form GABA by glutamate decarboxylase (GAD), and then converted to succinic semi-aldehyde under the transamination of GABA transaminase (GABA-T), which was finally decomposed into succinic acid under the action of succinic semialdehyde dehydrogenase (SSADH) located in the mitochondria [15]. Then, the succinic acid can enter the tricarboxylic acid (TCA) cycle. In polyamine degradation pathway, the oxidation of putrescine (Put), spermidine (Spd) and spermine (Spm) contributes directly to GABA accumulation [16]. The enzymes involved in the oxidative deamination of PAs are diamine oxidases (DAO) and polyamine oxidase (PAO), both of them catalyze Put, Spd and Spm to 4-aminobutyraldehyde (ABAL) and aminoaldehyde dehydrogenase (AMADH) thereafter to produce GABA [17,18]. Information about GABA accumulation in watercore fruit is limited. In the present study, the transcriptional changes in the TCA cycle, glycolysis metabolism and GABA metabolism in watercore pears was investigated to explore supporting evidence for GABA involved in pear water core prevention for the future.

## 2. Materials and Methods

### 2.1. Plant Materials

The watercore suspectively pear fruit, ‘Akibae’, was used in this study. During the fruit ripening period and about 125 days after flowering (DAF), ‘Akibae’ pear fruits were randomly selected from trees that had similar tree vigor with no plant diseases or insect pests. Pulp tissues were dissected and divided into healthy fruit and watercore fruit. The samples were immediately frozen in liquid nitrogen and stored at −80 °C for further use.

### 2.2. Measurement of GABA Content and GAD Activity

The GABA content was carried out using test kit of Suzhou Comin Biotechnology. About 0.5 g pear sample mixed with 5 mL extracting solution. After mixed well, it was heated in a water bath for 2 h at 95 °C. After cooling and centrifugation, the absorption readings were taken by a spectro-photometer at 640 nm. 

GAD activity was determined by test kit of Shanghai Enzyme-linked Biotechnology. About 1 g pear sample mixed with 9 mL 0.01 mol/L PBS for enzyme solution extraction. The enzyme solution mixed with standard solution reaction at 37 °C for 0.5 h, then added Elisa reagent reaction at 37 °C for 0.5 h, added color-substrate solution reaction for 10 min. The absorption readings were taken by a microplate reader at 450 nm. 

### 2.3. RNA Extraction and Sequencing

To evaluate the transcriptomic changes of key pathways response to watercore fruit, RNA-seq analysis was performed using watercore pulp tissues in three biological replicates. Samples RNA was isolated using the Trizol kit (Promega, MA, USA) following the manufacturer’s protocol. The cDNA library from each sample was sequenced on the Illumina HiSeq. The process refers to our previous work [5]. All RNA-seq data have been deposited in the National Center for Biotechnology Information database (Accession number: GSE164987). The high-quality clean data were mapped to the *Pyrus betulifolia* [19] genome. The differentially expressed genes (DEGs) were screened according to the following criteria: |log_2_foldchange| ≥ 1 and corrected at *p* < 0.05. 

### 2.4. qPCR Analysis

Gene-specific primers were designed using the software Primer5 (Table S1). Quantitative real-time PCR (qRT-PCR) was performed with a Bio-Rad CFX96 instrument (Bio-Rad, Waltham, MA, USA) using ChamQ SYBR qPCR Master Mix (Vazyme Biotechnology, Nanjing, China). The amplification procedures according to our previous study [5]. Gene relative expression levels was calculated by Livak’s [20] method.

### 2.5. Statistical Analysis

The data were evaluated by Excel. Differences were considered significant at *p* < 0.05. Graphs were drawn using the GraphPad Prism 7.0 scientific software (San Diego, CA, USA).

## 3. Results

### 3.1. Analysis of TCA Cycle-Related Gene Expression in Watercore Fruit

**Figure 1:**
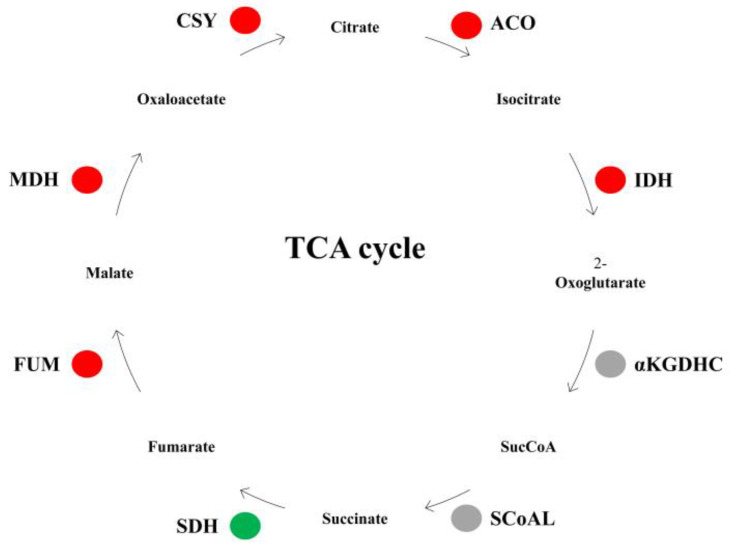
Changes of TCA cycle metabolism-related gene expression in watercore fruit. The red, green and gray circles refer to up-regulation, down-regulation and no changes of gene expression, respectively.

Analysis of DEGs found that two citrate synthase genes (Chr16.g30574 and Chr4.g39633), two aconitase genes (Chr9.g46434 and Chr15.g02728), two isocitrate dehydrogenase genes (Chr15.g01127 and Chr15.g02763), one fumarase gene (Chr3.g17593) and one malate dehydrogenase genes (Chr2.g43436) were significantly up-regulated in watercore fruit. Moreover, the expression levels of a succinate dehydrogenase gene (Chr10.g17307) was significantly decreased in watercore fruit. The αKGDHC and SCoAL genes had no significant changes (Table 1, Figure 1).

### 3.2. Analysis of Glycolysis Metabolism-Related Gene in Watercore Fruit

**Figure 2:**
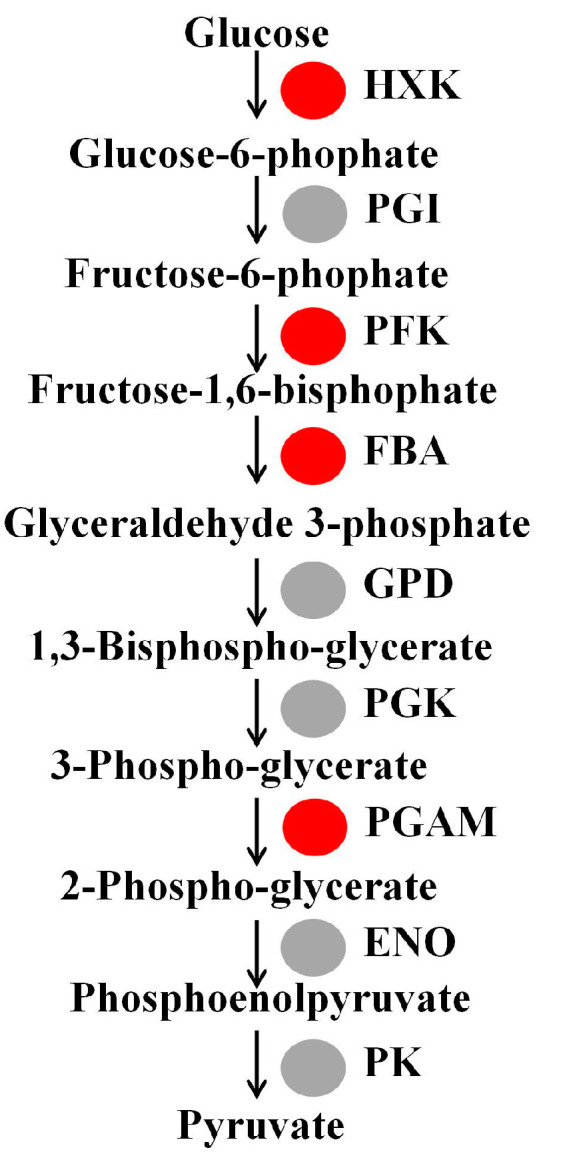
Changes of glycolysis metabolism-related gene expression in watercore fruit. The red and gray circles refer to up-regulation and no changes of gene expression, respectively.

Eleven genes involved in glycolysis metabolism were significantly up-regulated in watercore fruit. These genes included two hexokinase genes (Chr9.g45423 and Chr15.g04826), three phosphofructokinase genes (Chr17.g26626, Chr2.g41653 and Chr1.g58362), three fructose-bisphosphate aldolase genes (Chr11.g09985, Chr10.g16756 and Chr13.g23923) and three phosphoglycerate mutase genes (Chr14.g49092, Chr15.g02324 and Chr15.g02323) (Table 2, Figure 2).

### 3.3. Analysis of Genes to GABA Produced Pathway by GABA Shunt and Polyamine Degradation Related Gene Expression in Watercore Fruit

**Figure 3:**
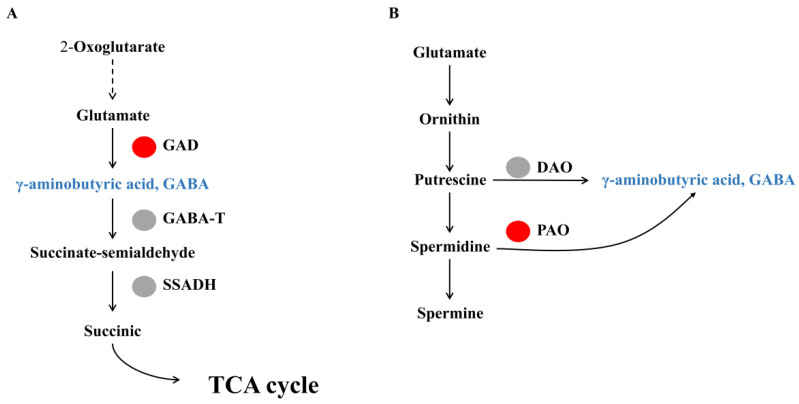
Changes of GABA shunt (**A**) and polyamine degradation (**B**) metabolism-related gene expression in watercore fruit. The red and gray circles refer to up-regulation and no changes of gene expression, respectively.

GABA shunt metabolism-related gene analysis showed that three Glutamate decarboxylase genes (Chr16.g31370, Chr9.g44430 and Chr6.g50750) were significantly upregulated. The expression levels of GABA-T and SSADH which were involved in GABA degradation, respectively, were not induced in watercore fruit (Table 3, Figure 3A). Moreover, two genes of polyamine oxidase (Chr7.g34714 and Chr9.g44410), which is GABA synthesis enzyme in polyamine degradation pathway, were significantly upregulated in watercore fruit (Table 3, Figure 3B).

### 3.4. GABA Content, GAD Activity and qRT-PCR Analysis of PpGAD Genes in Fruits at Different Developmental Stages or Watercore Fruit

**Figure 4:**
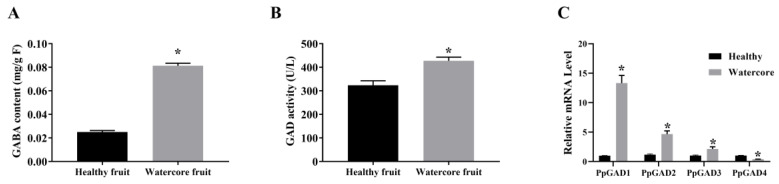
Analysis of GABA content (**A**), GAD activity (**B**) and PpGADs’ expression (**C**) in the watercore fruits. The asterisk on the bars represent standard errors from three independent replicates. Significant difference at *p* < 0.05.

As showed in Figure 4A,B, the GABA content and GAD activity were gradually increased in the fruits at different developmental stages. The four GAD DEGs, which were identified in Table 3 were named as *PpGAD1* to *PpGAD4* (Table S1). Transcripts of the four PpGAD genes were further detected by qRT-PCR, similar to the transcriptional changes. In detail, the expression level of *PpGAD1*, *PpGAD2* and *PpGAD3* in watercore fruit were over 13-, 4.6- and 2.3-times than those in healthy fruit. *PpGAD4* expression level was significantly decreased in watercore fruit (Figure 4).

## 4. Discussion

The accumulation of sorbitol in the intercellular spaces of affected fruit suggests an interruption of normal metabolism, because in healthy tissues sorbitol is converted to fructose by the sorbitol dehydrogenase enzyme before its utilization by cell respiration or starch synthesis. Yamada found that watercore-susceptible fruit have a more favorable water status for the accumulation of liquid in the intercellular spaces around the vascular bundle, which result in a higher incidence of early watercore. Thus, the watercore appearance was due to the filling of intercellular spaces with liquid, instead of air [21]. A long duration of water soaking is bound to cause fruit hypoxia. Hypoxic stress leads to metabolic changes. The most important and well-studied cases on this subject are those involving pyruvate metabolism, which switches from oxidative to fermentation metabolism, allowing for the reoxidation of NADH to maintain glycolysis and some ATP production. Here, pyruvate is transformed into lactate and ethanol, processes which regenerate NAD^+^ [22]. Upon hypoxia, the inhibition of mitochondrial oxidative phosphorylation triggers the ‘Pasteur effect’ leading to an increase in glycolysis to maintain ATP production and cell viability [23]. Other products of anaerobic metabolism can accumulate, including alanine and succinate. Hypoxia is known to determine a complete rearrangement of primary metabolism to ensure cell survival during oxygen shortage, by hijacking sugar metabolism from the TCA cycle, which is inhibited and reversed, toward an increased glycolytic flux. Ruperti et al. found in hypoxic grapevine roots the induction of the genes responsible for the conversion of CS, FUM and SDH, which results in the accumulation of succinate in response to hypoxia [24]. Succinic acid formation appeared to be further sustained through the activation of the gene encoding the alpha subunit of succinyl-CoA synthetase. Consistently with the inhibition of the TCA cycle, leading to acetyl-CoA shortage in hypoxic cells, fatty acids biosynthesis and elongation appeared overall inhibited [24]. In the present study, analysis the key genes of TCA cycle and glycolysis showed that the succinate was accumulated and the glycolysis pathway was enhanced in watercore fruit. Together with our previous research [4,5], it suggested that the over accumulated intercellular spaces sorbitol induced hypoxic stress leads to metabolic changes and accelerate fruit decay and poor flavor.

In plants, GABA can serve as an important nonprotein amino acid by providing a substrate for the TCA cycle and the electron transport chain. GABA formation is regulated by glutamic acid degradation catalyzed by glutamic acid decarboxylase [25]. GABA is initially synthesized and accumulates in the cytosol, after which it is transported to the mitochondria after being converted to succinic acid [26], which is the key intermediate linked to the GABA shunt and TCA cycle [16,27]. The GABA shunt is closely associated with increased tolerance to hypoxia in plants subjected to hypoxic stress, especially with respect to metabolite supplementation of the TCA cycle [24]. Besides, Wu et al. found hypoxia-induced increase in GABA content is essential for restoration of membrane potential and preventing ROS-induced disturbance [28]. Moreover, it has been suggested that GABA can be produced from polyamine degradation by DAO and PAO under abiotic stress [29,30]. In this study, We found most of the GAD and PAO genes were significantly up regulated and the GABA content was also significantly increased in watercore fruit. Because many studies confirms that low contribution ratio of polyamine degradation pathway for GABA formation, we further tested the GAD activity and *PpGAD* gene expression and we speculated that *PpGAD1* may play a vital role in the response to watercore induced hypoxic stress by being involved in the GABA synthesis. On the basis of research concerning endogenous GABA, studies have investigated the application of exogenous GABA and plant adaptations to hypoxic stress [31,32]. The results showed that exogenous GABA increased hypoxia tolerance of plants, which closely associated with marked increase in endogenous GABA. 

## 5. Conclusions

Water soaked induced hypoxia stress in pear fruits and their specific hypoxic response were marked by a significant increase of the hypoxia-inducible metabolites succinic acid, GABA and expression of several *PpGAD* genes. *PpGAD1* played a vital role in response to watercore. Exogenous GABA’s role in preventing of the occurrence of watercore will be further studied in the future.

## Figures and Tables

**Table 1:** List of DEGs related to TCA cycle metabolism.

Gene ID	log_2_^Fold Change^	Gene Description
Chr16.g30574	1.10	Citrate synthase, CS
Chr4.g39633	2.37	Citrate synthase, CS
Chr9.g46434	1.25	Aconitase, ACO
Chr15.g02728	8.70	Aconitase, ACO
Chr15.g01127	1.02	Isocitrate dehydrogenase, IDH
Chr15.g02763	1.46	Isocitrate dehydrogenase, IDH
Chr3.g17593	1.65	Fumarase, FUM
Chr10.g17307	−1.10	Succinate dehydrogenase, SDH
Chr2.g43436	2.07	Malate dehydrogenase, MDH

**Table 2:** List of DEGs related to glycolysis metabolism.

Gene ID	log2Fold Change	Gene Description
Chr9.g45423	1.04	Hexokinase, HXK
Chr15.g04826	1.84	Hexokinase, HXK
Chr17.g26626	1.02	6-phosphofructokinase, PFK
Chr2.g41653	2.38	6-phosphofructokinase, PFK
Chr1.g58362	1.71	6-phosphofructokinase, PFK
Chr11.g09985	1.19	Fructose-bisphosphate aldolase, FBA
Chr10.g16756	1.82	Fructose-bisphosphate aldolase, FBA
Chr13.g23923	3.90	Fructose-bisphosphate aldolase, FBA
Chr14.g49092	1.13	Phosphoglycerate mutase, PGAM
Chr15.g02324	6.70	Phosphoglycerate mutase, PGAM
Chr15.g02323	3.40	Phosphoglycerate mutase, PGAM

**Table 3:** List of DEGs related to GABA shunt and polyamine degradation pathway.

Gene ID	log2Fold Change	Gene Description
Chr16.g31370	6.45	Glutamate decarboxylase, GAD
Chr9.g44430	1.94	Glutamate decarboxylase, GAD
Chr6.g50750	1.51	Glutamate decarboxylase, GAD
Chr14.g50570	−2.64	Glutamate decarboxylase, GAD
Chr7.g34714	5.78	Polyamine oxidase, PAO
Chr9.g44410	1.08	Polyamine oxidase, PAO
Chr2.g41730	−3.88	Polyamine oxidase, PAO

## Data Availability

The RNA-Seq data of watercore pear fruit associated with this study were downloaded with NCBI GEO accession number: GSE164987.

## Supplementary Materials

The following supporting information can be downloaded at: https://www.mdpi.com/article/10.3390/genes13060977/s1, Table S1: Information of *PpGADs*.
