# Supplementary material for: Watercore Pear Fruit Respiration Changed and Accumulated γ-Aminobutyric Acid (GABA) in Response to Inner Hypoxia Stress
Source: Genes (Basel). 2022 May 30;13(6):977. doi: 10.3390/genes13060977 (PMC9222961; doi:10.3390/genes13060977)
Supplement: Supplementary file 1 [file genes-13-00977-s001.zip › genes-1709457-supplementary.pdf]

Table S1. Information of PpGADs

Gene ID	Gene Name	Primer List
Chr16.g31370	PpGAD1	F:AAATGGAAACGGCGAGTTGC<br>R:GTAGCATTTTGCACCGTCCG
Chr9.g44430	PpGAD2	F:TCTCTCGCACTTTGGCTGAG<br>R:CTCTCAAGTGTCGTCCCAGG
Chr6.g50750	PpGAD3	F:TGTCGACGAAGTAGTCAGCG<br>R:TGCCTAGCATACGCCTGTTT
Chr14.g50570	PpGAD4	F:CCGAGCCAAGTGTCTAGCA<br>R:CTCAACGTCTGTCACGCTCT
